# Facilitating Secure Sharing of Personal Health Data in the Cloud

**DOI:** 10.2196/medinform.4756

**Published:** 2016-05-27

**Authors:** Danan Thilakanathan, Rafael A Calvo, Shiping Chen, Surya Nepal, Nick Glozier

**Affiliations:** ^1^ Software Engineering Lab School of Electrical and Information Engineering The University of Sydney Sydney Australia; ^2^ Digital Productivity Flagship Commonwealth Science Industry Research Organization (CSIRO) Sydney Australia; ^3^ Brain and Mind Research Institute The University of Sydney Sydney Australia

**Keywords:** self care, telemedicine, privacy, computer security, information dissemination

## Abstract

**Background:**

Internet-based applications are providing new ways of promoting health and reducing the cost of care. Although data can be kept encrypted in servers, the user does not have the ability to decide whom the data are shared with. Technically this is linked to the problem of who owns the data encryption keys required to decrypt the data. Currently, cloud service providers, rather than users, have full rights to the key. In practical terms this makes the users lose full control over their data. Trust and uptake of these applications can be increased by allowing patients to feel in control of their data, generally stored in cloud-based services.

**Objective:**

This paper addresses this security challenge by providing the user a way of controlling encryption keys independently of the cloud service provider. We provide a secure and usable system that enables a patient to share health information with doctors and specialists.

**Methods:**

We contribute a secure protocol for patients to share their data with doctors and others on the cloud while keeping complete ownership. We developed a simple, stereotypical health application and carried out security tests, performance tests, and usability tests with both students and doctors (N=15).

**Results:**

We developed the health application as an app for Android mobile phones. We carried out the usability tests on potential participants and medical professionals. Of 20 participants, 14 (70%) either agreed or strongly agreed that they felt safer using our system. Using mixed methods, we show that participants agreed that privacy and security of health data are important and that our system addresses these issues.

**Conclusions:**

We presented a security protocol that enables patients to securely share their eHealth data with doctors and nurses and developed a secure and usable system that enables patients to share mental health information with doctors.

## Introduction

A new type of sociotechnical challenge has arisen with the advent of eHealth and big data technologies. For example, ubiquitous and wearable health systems collect data through sensors and mobile apps and store the data in the servers of multiple commercial service providers. Furthermore, a growing number of people share this sensitive medical information through social networks such as Facebook and Twitter. This is significantly different from the traditional health service, where service providers kept tight control over patient data.

It has been argued that these new technologies can lead to positive health outcomes, as they are evidence of people self-managing their illness [1]. Some of the ways in which self-management can have a positive effect include supporting the patient’s motivation to look after their health, greater levels of engagement, and understanding about the condition.

Furthermore, these new technologies may help improve population health by helping researchers learn about the drivers of different pathologies, or how people's behavior is affected by social influence and public health promotion campaigns [2]. The information posted to social networks can prove invaluable in assisting doctors and counselors to better understand patient behaviors and symptoms and can help to provide support and/or consultation. Social networks are now being leveraged to provide people with a better lifestyle and health, without the need to continually visit the doctor’s clinic.

However, privacy [3], trust, and security issues associated with health data make patients hesitant to post sensitive health information and share it with health providers [4]. Data are not ephemeral and will be stored in servers and shared. All stakeholders need to worry about the lifecycle of the data; not just who can access and manage the data at a particular point in time, but also who will be able to do so in the future. There is a strong need to provide patients with a guarantee that their sensitive health information will only be visible to the doctors, counselors, or others they wish to share it with at a particular point in time.

A trivial solution to sharing data in the cloud involves the data owners first encrypting their data before storing to cloud servers. The data owner can then distribute encryption keys to every user in the group thereby keeping the data protected from the cloud provider and also malicious users. Authorized users in the group can then download the encrypted data from the cloud and decrypt the data using the encryption key provided. However, the main problem with this solution is user revocation. When the data owner wishes to revoke one of the users in the group, he must re-encrypt the data with a new encryption key and redistribute the new key to all the remaining users in the group. This renders the revoked user’s key useless and he or she will thus not be able to access the data contents. This process of re-encrypting the data and redistributing keys to all the remaining users in the group every time a user is revoked access can place a huge burden on the data owner. This is especially the case when the group size is very large, in excess of thousands to hundreds of thousands (eg, everyone in an organization or online community).

There is a growing body of research on the trust, privacy, and security in information systems, most of which apply to health.

### Trust and Privacy

These issues often arise from insider attacks. For example, malicious insiders to a cloud service provider (eg, employees) can steal data, because they have direct access to it. Insiders who are not happy with their job and who have recently been terminated may take revenge and destroy, corrupt, or sell all data owner’s data [5]. Organizationally, cloud service providers may misuse data in order to sell to third parties [6,7]. Such privacy attacks affect the trust of users and make them skeptical of using cloud services for sensitive data storage. It has been argued that this is one of the main reasons why patients have a lack of trust for using the cloud for storage and sharing of highly critical medical information [8,9].

There have been multiple studies around privacy and trust in health systems in research [10-15]. One of the most effective ways of keeping data private in the cloud, and thus increasing the trust of the data owners, is keeping data encrypted when stored on untrusted servers, backup servers, and when in transit on untrusted public channels.

The THEWS (Trusted eHealth and eWelfare Space) architecture [16] provided privacy management to help data owners create and manage the network as well as maintain information privacy. As Ruotsalainen et al [16] pointed out, there is an asymmetric relationship between health information systems and their users because users rarely have the power “to force a system to put personal rules into effect.” Our paper contributes a novel security architecture that can help balance this power difference.

Even when data are encrypted, it may still be possible for a malicious cloud provider to deduce information from the encrypted data. Zhang et al [17] propose a novel solution that adds noise obfuscation based on a time-series pattern to client data stored in the cloud. This can help protect the privacy of the owner’s data because it prevents malicious service providers from deducing information from the encrypted data.

Little of this work has focused on private data sharing between patients and doctors using social networks. We present a new security model that would allow users to have a much more fine-grained control of their health data.

### Security

One of the major issues with private sharing of health information, and hence the major focus of this paper, is encryption key management. As discussed above, the trivial solution is computationally inefficient when having to revoke users because of the burden on re-encryption and redistribution of keys.

Microsoft HealthVault [18,19] provides a next step to allowing patients to store and manage their health and fitness information, as well as share the data securely with their friends and family. The encryption is done within HealthVault and does not rely on the patient to generate and distribute keys. The patient can decide who specifically can view his health information. With our system, the patient has greater control over his health information and can choose to store his health data on any cloud service provider that he wishes. The patient himself distributes encryption keys to people he wishes to share the data with and does not rely on commercial services, which may be untrustworthy.

Proxy re-encryption and attribute-based encryption (ABE) [20] are two current techniques aimed at secure and private data sharing in the cloud [21]. Ming et al [22] use ABE for efficient revocation for outsourced data sharing control. Liang et al [23] combine ABE with proxy re-encryption to achieve stronger security.

Silva et al [4] present a data encryption solution for mobile health apps and a performance evaluation comparing both symmetric and asymmetric encryption algorithms. Our work takes advantage of both symmetric and asymmetric cryptographic algorithms to achieve both strong security and high performance eHealth data using mobile phones.

### Other Related Work

Tran et al [24] utilize the idea of a proxy re-encryption scheme where the data owner’s private key is divided into two parts, where one is stored in the data owner’s machine and the other on the proxy. We also use this concept in our work and apply it to data sharing with many users instead of just one user.

Huda et al [25] propose a privacy-aware patient-controlled personal health record system that provides the patient the ability to control who can access which part of the patient’s health record as well as view health history. A shared key is used to control data access. In our work, we send key partitions to doctors as this allows for more efficient consumer revocation. We also use mobile apps because of their increased popularity.

In our previous work [26], we focused on secure sharing of electrocardiographic (ECG) data using a sensor, mobile phone, and the cloud. The sensor connects to the mobile phone via Bluetooth and streams encrypted ECG data to the cloud. Like Tran et al [24], we use a form of proxy re-encryption where keys are partitioned and shared with other doctors. Revoking a user would simply involve removing the corresponding doctor’s key partition in the cloud.

Furthermore, we applied our key partitioning encryption solution in two studies [27,28]. In one [27], we developed a software object that will carry out background monitoring to hold data consumers accountable if they breach the policy set out by the data owner. In the other [28], we applied our solution to a big data analysis in the health domain.

Our work leverages existing encryption algorithms to help build a more secure protocol that allows health data to be shared between a patient and many doctors, where the patient is in full control over who can access his health data and who cannot.

Our contribution is a new way of protecting data, without revealing the full encryption key to both the user and the cloud provider. The encryption key is a string of digital information that defines what a cryptographic algorithm produces, that is, how data are encrypted/decrypted. This is in addition to users’ passwords. The encryption key is used to generate a ciphertext of the original data and hence make the data illegible to ordinary users. The encryption key is used to decrypt or convert the ciphertext back to the original plaintext data.

We propose a system that is designed to be highly scalable, providing the ability to share data with many users, such as doctors and nurses, while allowing the simple revocation of a user without the need to re-encrypt the data every time a user revocation occurs. We focus on creating a secure and usable system that will enable patients to share mental health information with doctors and mental health specialists, from the comfort of their own home.

In this project, we evaluate the security model through a prototypical mobile phone app. We chose to recruit students and medical professionals to evaluate the security of our system because they were the most likely potential users of the system. Using a mobile phone app, patients can report and receive help, wherever they are. In the field of mental health, for example, studies have also shown that the use of mobile phone apps can support significant reductions in depression, stress, and substance use [29].

## Methods

Our system is built upon a requirements-driven design methodology [30].

**Figure 1:**
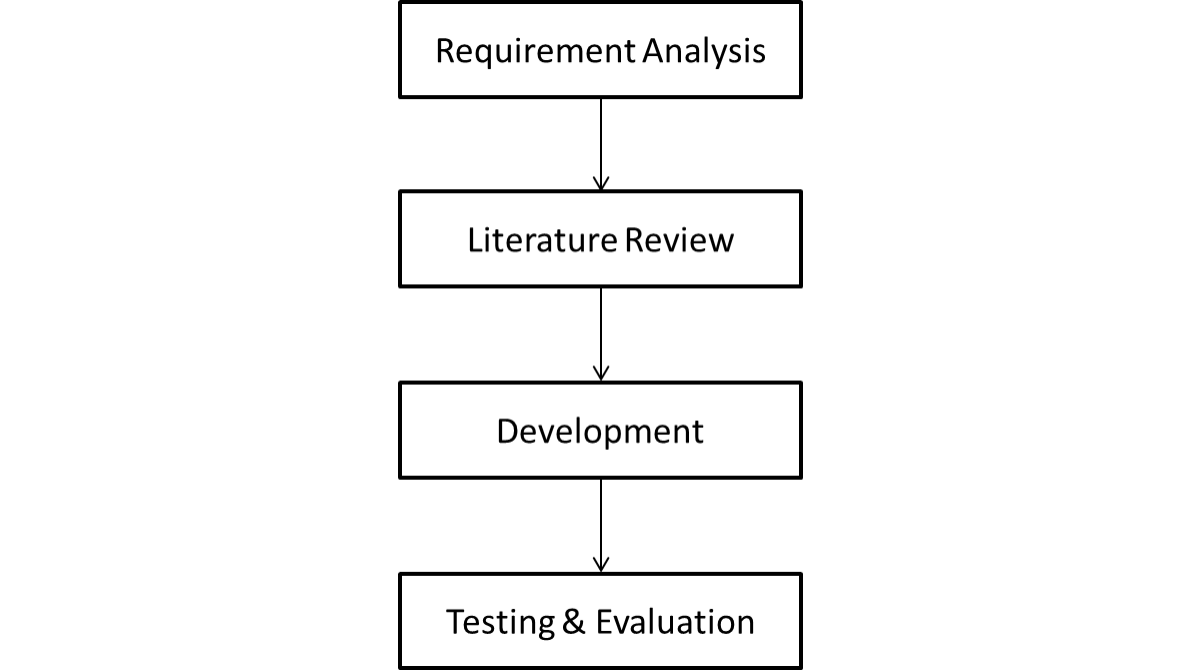
Development Method.

Figure 1 highlights the methodology we used to carry out our work. We first define the requirements of our work. That is, to develop a system that allows patients to share their personal health information securely and privately, while ensuring the system is usable. We use a fictitious scenario to assist in defining the requirements of the system. We then review state-of-the-art literature to explore the existing works or technologies that attempt to address this. We then build on these works and develop new technology. Finally, we test our developed system through performance and scalability tests and evaluate the system in terms of usability.

The secure encryption protocol has been developed over several projects at the Commonwealth Scientific and Industrial Research Organisation [21,26,28]. We also use the key partitioning technique in this work through the existing ElGamal encryption algorithm [15] because it is most suitable for efficient user revocation. In this paper, we leverage the key partitioning technique and mobile phones to provide patients with a new way of sharing their personal health information with doctors anywhere anytime while having the ability to control which doctor is able to access that information.

For the evaluation discussed in this study, we created a fictitious, but quite common, scenario: collecting data and providing support.

The best way of designing and then evaluating a security feature is through a minimum viable application in a realistic scenario. This security feature would be applicable in other scenarios, but the reification into concrete terms with users, and evaluate the design on scalability and nonfunctional requirements. Our application emulates one where data are collected to provide support to people at risk of mental health issues at the workplace.

We chose this scenario because it was relevant to our research and because of its significance. There is evidence of increased work stress, sleep disorders, and depression in the workplace [31]. As a result, there is a need for the means through which an organization can provide support and feedback in a convenient and secure manner. In order to detect people at risk, information is needed. This information may come from the people themselves or their friends, reporting problems at home or at work that are affecting their lives and their mental state. It could also come from managers, occupational health and safety reports, or other sources such as other eHealth systems. Regrettably, in many cases, people fail to seek help when they need it because of a number of reasons, including the lack of time or access to resources, stigma, and trust. For example, regularly visiting a clinic can be costly for patients and doctors. For patients, this also involves the time and effort spent visiting the clinic, particularly for rural and disabled patients. For doctors, eHealth may allow them to prioritize differently and tend to patients who cannot travel. Others have highlighted the possibility of using eHealth services to reduce health care costs [32].

We also speculated that certain aspects, characteristic of mental health issues, would make the importance of trust and privacy more relevant to users. Trust and stigma also make it harder for people to seek help or share information about their mental health. In workplace well-being programs, for example, employees might be less likely to share information if they feel that it could be used by their employers. Trust is in great measure a consequence of the software design of systems and apps used to collect and manage the data.

### Preliminaries

#### ElGamal Cryptography

We take advantage of ElGamal encryption [15], a public-key cryptographic system with an algorithm that is both simple and efficient and can provide simple consumer revocation with a low cost and overhead. ElGamal encryption, invented by Taher ElGamal [15], is a public-key cryptography system. One of the drawbacks of ElGamal encryption is that it is very computationally inefficient and time-consuming to decrypt fairly large data. Thus, the algorithm is best suited to the encryption and decryption of small data. In this project, we mainly use ElGamal encryption to add a further layer of protection, by encrypting/decrypting another encryption key instead of the data.

There are three main steps of the ElGamal encryption algorithm:

- Initialization: Given a prime p, a primitive root c of p, compute b=c^x^mod p, where x is a randomly selected secret key. The public key is thus {p, b, c} and private key is x.
- Encryption: Generate random value r and encrypt data m as follows:

E(m) = m×b^r^mod p=m×c^rx^mod p

Also note: g=c^r^mod p

Decryption: This decrypts m with secret key x as follows:

D_x_(E(m)) = g^-x^×E(m) mod p=(c^r^)^-x^×m×c^rx^mod p=c^-rx^×m×c^rx^mod p=m mod p

#### Symmetric/Asymmetric Cryptography

We use both symmetric and asymmetric encryption and decryption in our work to protect the health data from being accessed by untrusted social networks. We utilize both cryptography methods because they provide stronger security and higher performance while supporting larger data sizes in eHealth.

### Architecture

**Figure 2:**
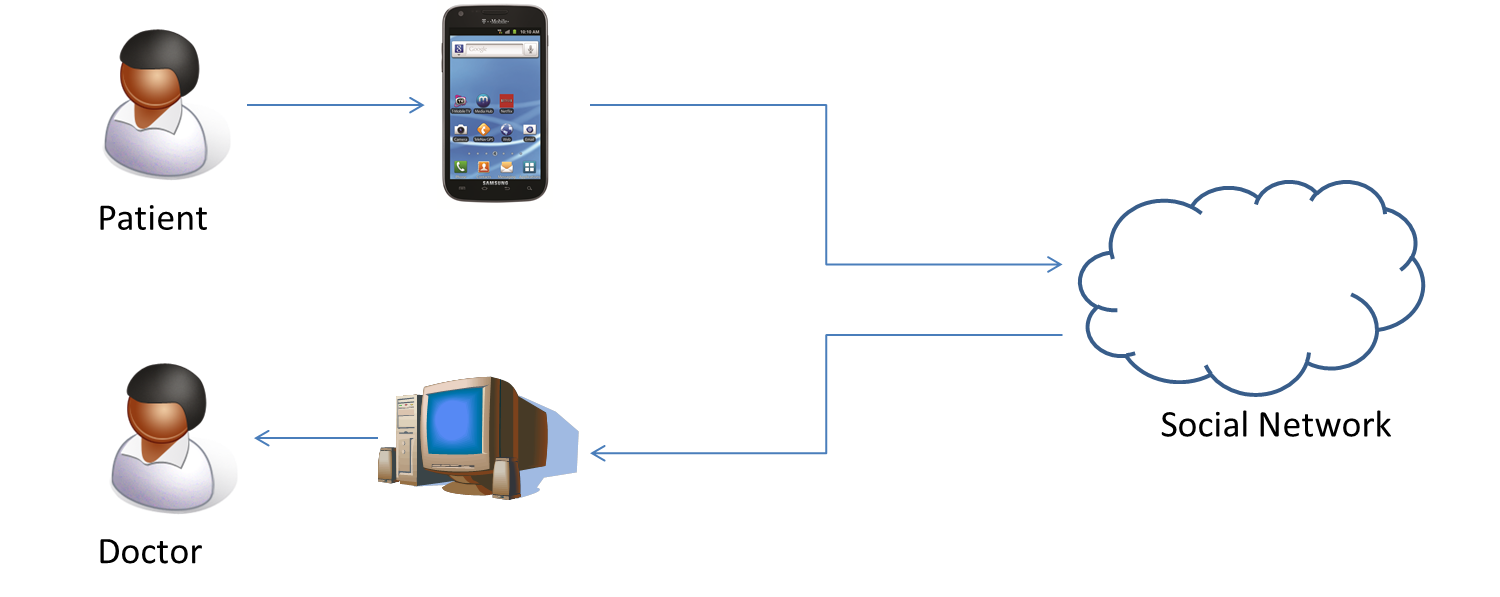
User Interactions in the System.

Figure 2 demonstrates our system. The model we used to test our application assumes a patient who monitors and tracks their health and activity data through a mobile phone app. The app may then connect to, and store the data in, a social network such as Facebook, Fitbit, or other cloud-based service provider using an application programming interface. An authorized doctor can log in to and retrieve the patient’s data and use the data for analysis and diagnosis.

For the sake of our evaluation, we have simplified the application so that it provides the most common features found in commercial products. Our prototype app allows the patient to enter a text value (eg, the description of an activity), a number value (eg, the amount of time spent), and an image. The app also includes a button used to encrypt the text, number, and image and send the data to a cloud server that is used to represent the social network. In our work, we developed a local cloud server that does encryption/decryption operations.

One of the main limitations of our work is that current social networks cannot automatically carry out encryption/decryption. However, we mainly wanted to demonstrate the potential capability of our system should a social network provide this feature in the future. Another limitation of our work is that, once the doctor has fully decrypted the patient’s health data, there is no way to revoke access. This is currently beyond the scope of our paper. The doctor however, would not be able to view any further health information posted by the patient.

One of the main goals of our system is to make it simple to use for both patients and doctors. Our system is not designed to replace existing health record systems but provide a convenient way for patients and doctors to communicate with each other remotely while ensuring privacy and security of health data. In terms of privacy, we offer a solution that enables the patient to define who can access their personal health data. We do not focus on the other aspects of privacy such as determining when the data were accessed, how the data were accessed, and to what extent the data are communicated. In terms of security, we provide solutions to availability through the use of the cloud and confidentiality in terms of allowing only authorized doctors to access the data. We do not focus on integrity or accountability in this work.

### Protocol

To describe the protocol, we assume that the patient’s public and private key pair has already been generated and stored in the app. We also assume the social network to be honest-but-curious in the sense that the rules of the protocol will be followed as intended but will still try to find out any sensitive information if possible.

#### Data Storage

**Figure 3:**
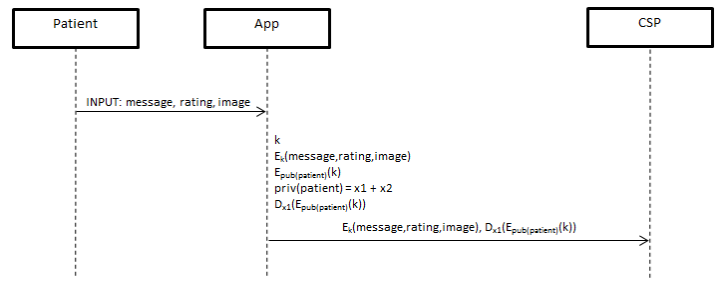
Data Storage Protocol.

The patient first runs the prototype app and inputs a text string and a number value, and uploads an image onto his mobile phone. When the patient presses the “Send” button, the app will then generate an arbitrary symmetric key and encrypt the text, number, and image. The symmetric key will then be encrypted using the public key. The encrypted data contents and encrypted symmetric key will then be sent to the social network, for storage (see Figure 3).

#### Data Sharing

**Figure 4:**
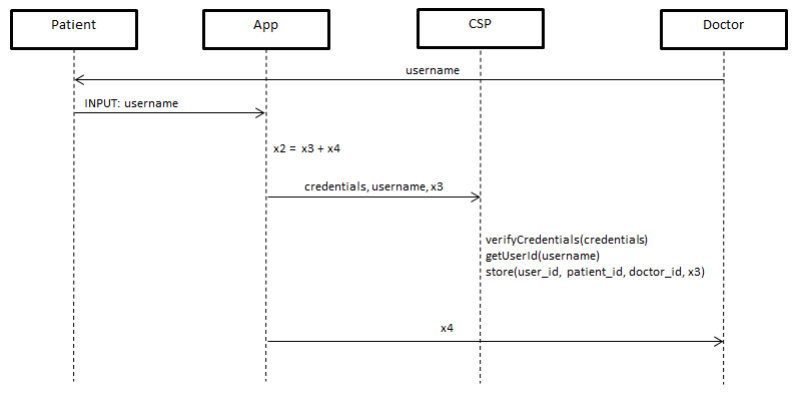
Data Sharing Protocol.

When the patient decides to share the data with a doctor, he presses the “Share” button on the app and enters the doctor’s social network username. The app will then partition the patient’s private key into 2 random parts. The first partition will be sent to the social network and the other will be sent to the doctor. By doing this, the untrusted social network has no knowledge of the full private key, because the other partition is stored on the doctor’s local machine (see Figure 4).

#### Data Access

**Figure 5:**
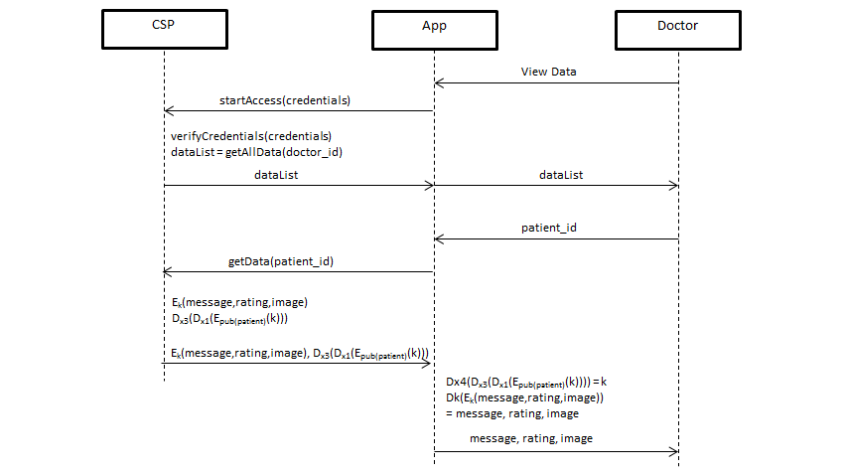
Data Access Protocol.

When the doctor wishes to access the patient’s data, they simply call the social network to retrieve the data. The social network partially decrypts the symmetric key using the partial key supplied by the patient and sends the encrypted data contents and partially decrypted symmetric key to the doctor. The doctor uses the partial key supplied by the patient to fully decrypt the symmetric key and finally decrypt the data contents. The standard method of accessing data involves the data consumer downloading the encrypted data from the cloud and decrypting the data on his own machine, using the encryption key supplied by the data owner. In our protocol, the data consumer does not have access to the other half of the key, which prevents the data consumer from ever knowing the full encryption key (see Figure 5).

#### Access Revocation

**Figure 6:**
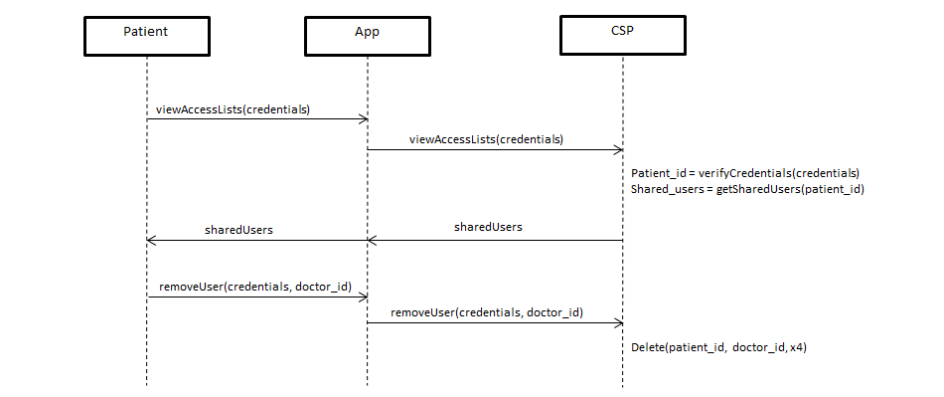
Access Revocation Protocol.

When the patient decides to revoke a specific user’s access to his eHealth data, the patient sends a request to the social network platform to remove the doctor’s partial key entry from storage. If the doctor attempts to download the data from the social network, he will only see the encrypted text (“ciphertext”). The doctor will not be able to fully decrypt or read the data without the partial key. In the trivial solution described earlier, the data owner would have to re-encrypt the data and redistribute the new encryption key to all of the remaining consumers in the group, thus placing a burden upon the data owner. In our solution, because the data consumer has no knowledge of the other half of the key partition stored in the cloud, the data owner would simply have to delete that key partition. Thus, he need not worry about re-encryption and the redistribution of keys (see Figure 6).

### Security Analysis

To verify the security of our protocol, we have used an automatic cryptographic verifier tool called ProVerif [33], which has been used extensively in research work [34].

We first modeled the behavior of the symmetric and asymmetric encryption, ElGamal encryption/decryption, and digital signatures.

We then modeled the patient by following the logic of the protocol. In other words, the patient sending their encrypted health data to the cloud server is modeled.

The cloud provider model simply retrieved the encrypted data from the patient via the public communication channel. When requested by the doctor, it would carry out a partial decryption of the symmetric key using the doctor’s key partition and send it back to the doctor via the public communication channel.

The doctor was modeled as retrieving the key partition from the patient via the private communication channel. The doctor then retrieves the encrypted data and uses her own key partition to fully decrypt the partially decrypted symmetric key and then fully decrypt the encrypted data to reveal the plaintext health data.

Each of the processes of the data owner, cloud provider, and data consumer were run simultaneously, to simulate realism.

### Usability Analysis

#### Participant Recruitment

In total, we recruited 5 medical professionals and 15 students to carry out the usability testing of our eHealth application. According to Nielsen [35], the minimum number of participants required in a usability study is 5. We chose to recruit medical professionals, because of their experience with patients and health issues. They were also the most likely potential users of our system. The medical professionals included 2 doctors, 2 medical officers, and 1 medical intern. We chose also chose young people (ie, students) because they were the most likely to use mobile phones and would be likely potential end users of the system. We recruited students aged more than 18 years. Of the 20 participants, 17 (85%) were aged more than 25 years and 3 (15%) were from 18 to 25 years of age. We obtained ethics approval to carry out the study. All students reported having a fair amount of experience using mobile apps.

To carry out the usability tests, we provided participants with a 4-inch LG mobile phone with Android operating system (OS) and a 10-inch ASUS Eee Pad tablet [36], which contained our secure eHealth app. We also launched our Web service, which would interact with the mobile phone to store and retrieve eHealth data and enable the sharing with, and revocation of, other users.

All 20 participants were given the same demo. Each participant was first introduced to the main idea of our secure eHealth system. We then asked the participants to carry out simple tasks such as the following:

- Report current mood
- Share information with another user
- Show that the other user can view the user's mood submission
- View mood submissions, etc

Each participant was told that their mood submission was encrypted, and they were shown the back end of their stored mood submission. Participants then answered our trust and usability questionnaire.

**Figure 7:**
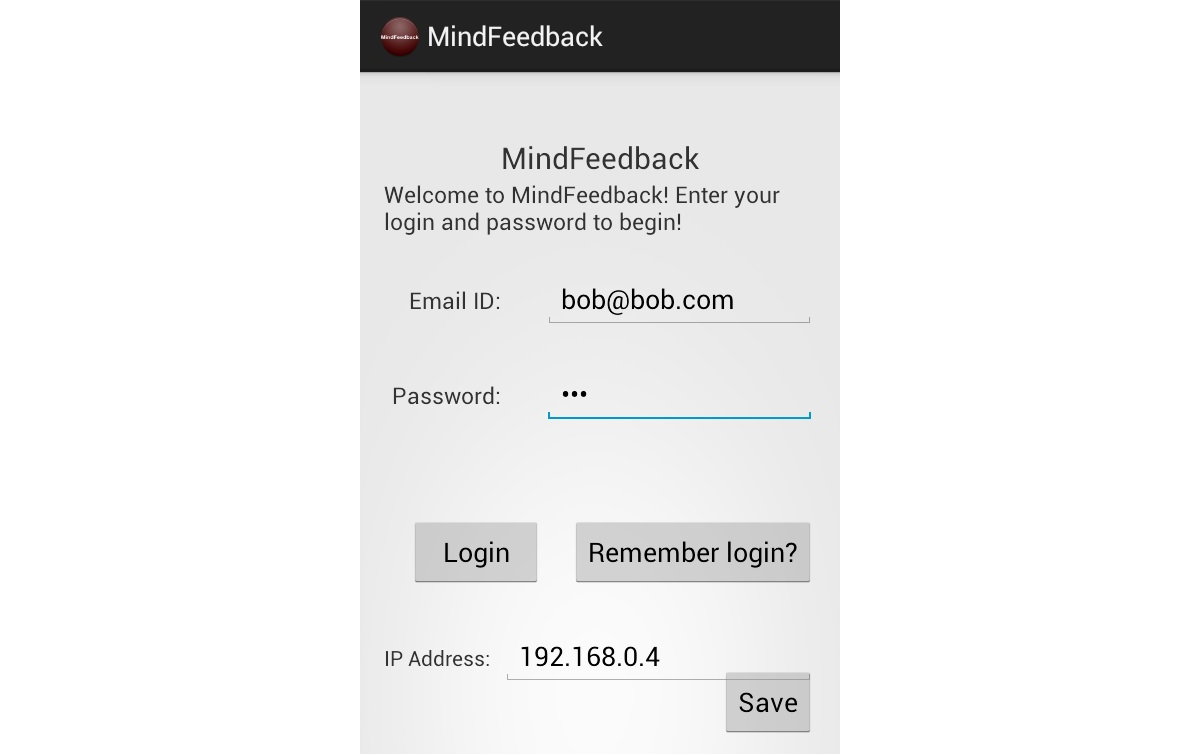
Screenshot of app login.

**Figure 8:**
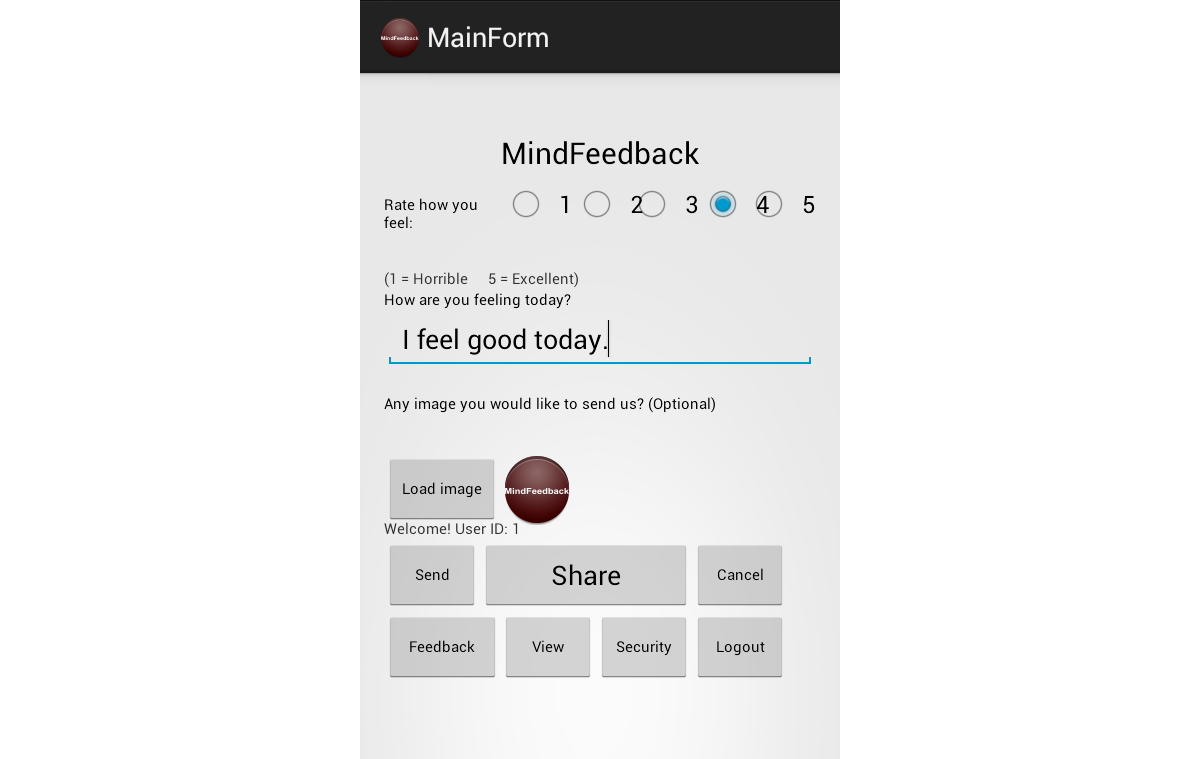
Screenshot of patient mood input.

**Figure 9:**
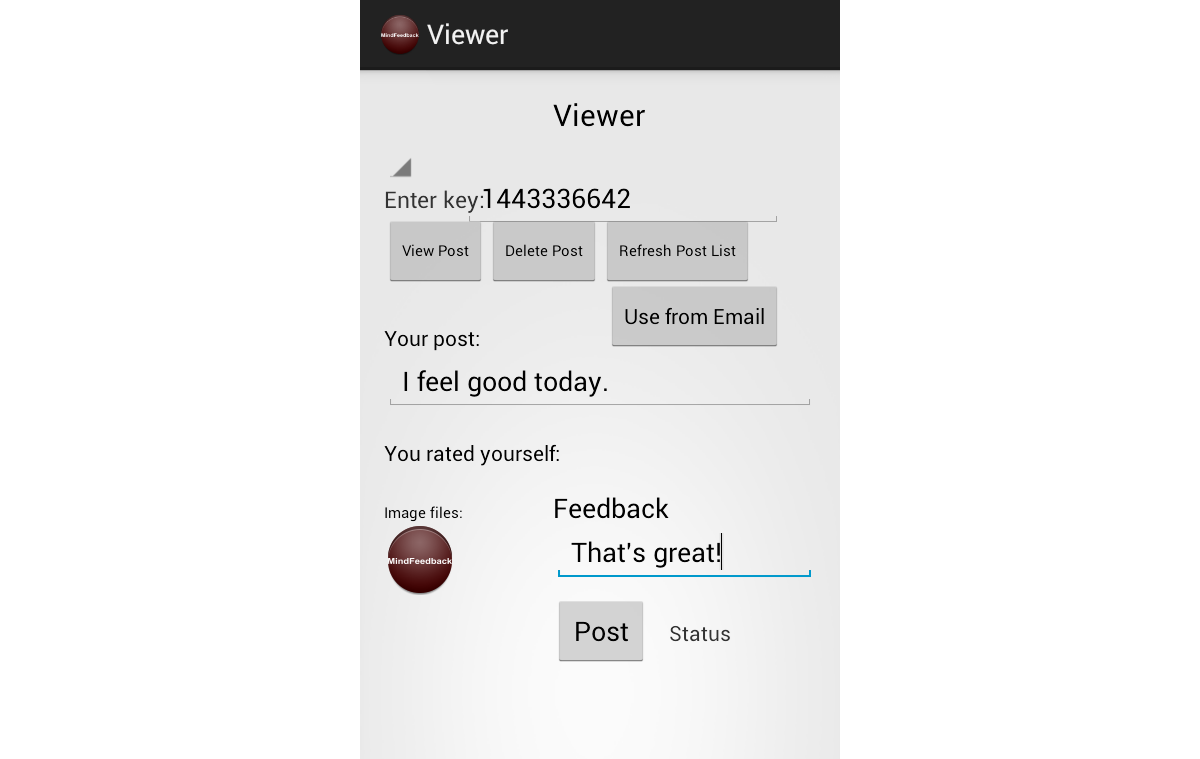
Doctor's view of patient's health data.

We have illustrated the user interface of our MindFeedback app with Figures 7-9.

#### Instruments

We asked the participants to think aloud while taking notes. Finally, participants answered a short questionnaire (see Textbox 1), with questions related to trust and security [11,12] and usability (the Usability, Satisfaction and Ease of use questionnaire [37]). The questionnaire asked the participant to assess our system based on trust and security, ease of use, and satisfaction, based on a 7-point Likert scale.

We investigated the relationship between how trustworthy and secure our system is and how useful our system is to everyday users. SurveyMonkey was used to provide the questionnaires to the participants and to carry out the analysis of the questionnaire responses.

**Questionnaire for all participants**

Demographic questions

  Are you male or female?

  What is your ethnicity?

  What is the highest level of school you have completed or the highest degree you have received?

Seven-point Likert scale questions

Trust and security

    When I’m connected to the Internet, I am concerned about exposing my health information to the public.

    I am not too concerned about what others see when I post my health-related information on the Internet.

    This system has made me more aware of what I may be exposing to others on the network.

    I feel safer when using the system.

    Personal information, which I input, is managed carefully and will not be leaked.

Ease of use

    It is easy to use.

    It is user-friendly.

    It requires the fewest steps possible to accomplish what I want to do with it.

    Both occasional and regular users would like it.

    I can use it successfully every time.

    The app is tedious.

    I require written instructions to use it.

    It is difficult to recover from mistakes.

Satisfaction

    I am satisfied with it.

    It works the way I want it to work.

    The app could be better.

    The app wasn’t as satisfactory compared to other health apps.

Feedback

    Would you like to provide any other feedback on our system?

### Performance Tests

The computational overhead introduced by our encryption system on storage and retrieval of eHealth information was tested with simple Advanced Encryption Standard (AES) encryption/decryption of similar text data. We carried out 20 test cases and measured the time taken for each test case. To carry out the tests, we used the ASUS Eee Pad Transformer Prime TF201 tablet with Android OS [36] to run our MindFeedback app. Testing was done on an HP Notebook running Windows 8 with Intel Core i5 and 4GB RAM to run the AES encryption/decryption operations and also to interface with our app to retrieve performance time information of MindFeedback.

### Scalability Analysis

The scalability tests measured the maximum load distribution our system can handle. This was done using a commercial scalability testing tool that made calls to the login() and getdata() methods of our cloud service. The tests showed that the maximum number of threads executed concurrently without the system becoming a bottleneck was 200. Tests were on an HP Notebook running Windows 8 with Intel Core i5 and 4GB RAM.

## Results

### Security Analysis

#### Informal Analysis

We now provide a brief security risk analysis of our work.

- Insider attacks: Our protocol prevents insider attacks because the data are never fully decrypted in the untrusted cloud under any circumstance. The data remain encrypted at all times on the untrusted cloud servers as well as on untrusted public communication channels.
- User revocation: Revocation of a doctor from data access can be achieved efficiently without having to re-encrypt the data each time. The doctor’s key partition is simply removed from the cloud storage. This way, if the revoked doctor now attempts to access the health data, he will not be able to retrieve the full plaintext without the remaining key partition.
- Update secrecy: Because health data are constantly changing, patients may wish to update their health data. This is made possible in our protocol; as long as the updated version is encrypted with the same symmetric key that was used to encrypt the original health data, the patients may update their health data any number of times as they wish. This makes our solution feasible to be deployed in a real-world scenario.
- Mobile stealing: In the event someone steals the patient’s mobile phone, they will not be able to access the personal health information as they would need to know the patient’s credentials such as email id and password in order to access the mobile phone app. Hence, a patient does not need to be tied down to only one mobile phone device and can keep changing his device as often as he would like without any loss of personal health information.

#### Formal Analysis

We used an automatic cryptographic verifier tool called ProVerif [33] to formally verify our protocol. The tool tests the protocol against all types of adversary attacks, such as man-in-the-middle attacks. We tested the storage of eHealth data by the patient and the retrieval of health data by an authorized doctor. Specifically, we tested the Data Storage and Data Access phases of our protocol. Our protocol was found to be secure against such attacks.

**Figure 10:**
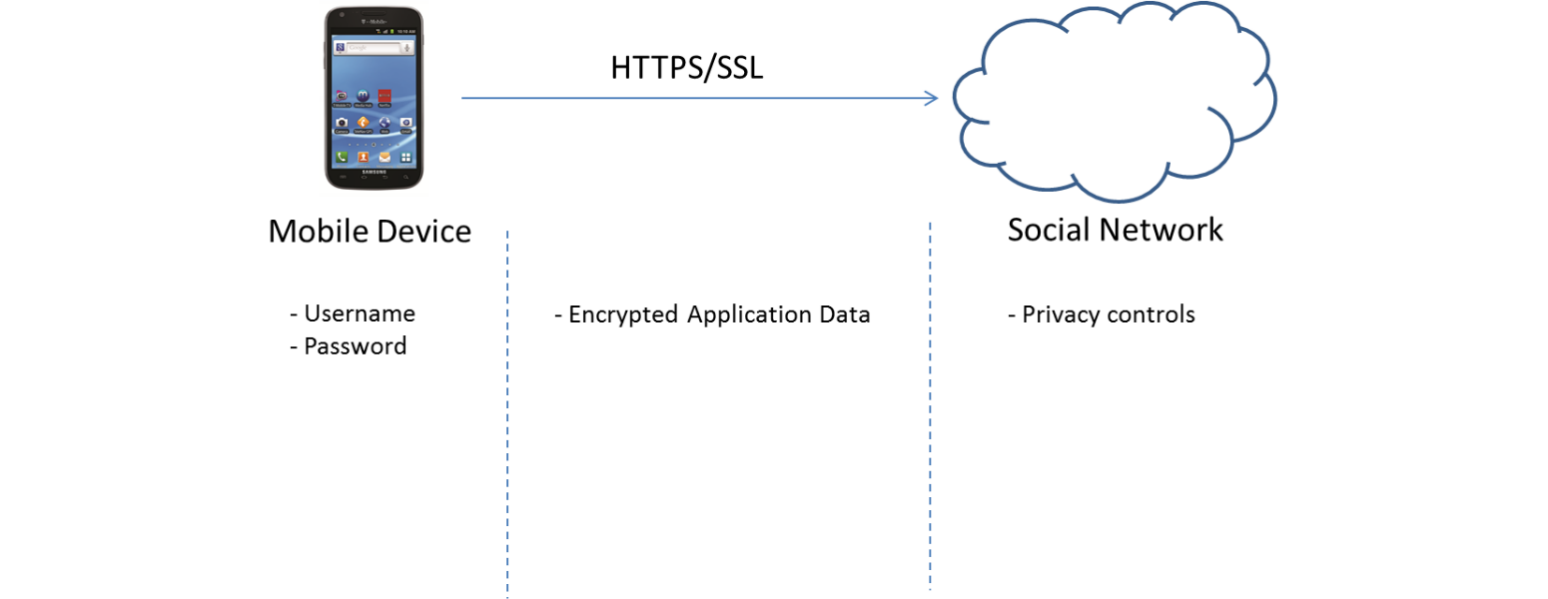
Secure communication paths.

Figure 10 illustrates the security mechanisms used in our system. The mobile app requires username and password credentials to be able to use our system. All health data that are sent to the social network are encrypted and sent securely via HTTPS/SSL. The social network also has privacy controls that the patient can adjust to suit their needs.

### Usability Analysis

#### Potential Users

We conducted a quantitative-based usability evaluation. Table 1 contains the responses from the 15 participants regarding the questions related to trust and security, ease of use, and satisfaction.

From our trust and security results, 10 out of 15 participants (67%) had at least some concern over what others see when they post health-related information on the Internet. Out of 15 participants, 12 (80%) felt that their data would be kept private and secure when using our system and were also made more aware of the type of information that they may be exposing over the Internet. In regard to whether their personal information will be managed carefully and not leaked to the outside, nearly half of the participants agreed. Participants did mention that some form of training or a video demonstration would have communicated the security of the system a lot more effectively.

From our ease-of-use responses, we found that 11 out of 15 participants (73%) found our system easy to use and learn, user-friendly, and were able to use it successfully, every time. However, 4 out of 15 participants (27%) did find the app a little “tedious” to work with initially, and required some instructions to understand the system a little better. Overall, the satisfaction of the app was mostly positive. Out of 15 participants, 13 (87%) were satisfied with our app and found that it worked in the way they wanted it to. However, most agreed that the app could have been improved. For instance, participants provided feedback that the app could have had a better-looking and much more intuitive interface.

**Table 1:** Potential users responses to questionnaire

Question	Strongly disagree	Disagree	Partially disagree	Neither disagree nor agree	Partially agree	Agree	Strongly agree
**Trust and security**							
When I’m connected to the Internet, I am concerned about exposing my health information to the public.		2 (13.33%)	2 (13.33%)	1 (6.67%)	2 (13.33%)	5 (33.33%)	3 (20.00%)
I am not too concerned about what others see when I post my health-related information on the Internet.	4 (26.67%)	5 (33.33%)	1 (6.67%)	2 (13.33%)	2 (13.33%)	1 (6.67%)	
This system has made me more aware of what I may be exposing to others on the network		2 (13.33%)		2 (13.33%)	1 (6.67%)	8 (53.33%)	2 (13.33%)
I feel safer when using the system.		1 (6.67%)	1 (6.67%)	1 (6.67%)	2 (13.33%)	7 (46.67%)	3 (20.00%)
Personal information, which I input, is managed carefully and will not be leaked to the outside.		2 (13.33%)	1 (6.67%)	2 (13.33%)	3 (20.00%)	5 (33.33%)	2 (13.33%)
**Ease of use**							
It is easy to use.		1 (6.67%)	2 (13.33%)	1 (6.67%)		8 (53.33%)	3 (20.00%)
It is user-friendly.		1 (6.67%)	1 (6.67%)	2 (13.33%)	1 (6.67%)	7 (46.67%)	3 (20.00%)
It requires the fewest steps possible to accomplish what I want to do with it.		2 (13.33%)		2 (13.33%)		9 (60.00%)	2 (13.33%)
Both occasional and regular users would like it.		3 (20.00%)	1 (6.67%)	1 (6.67%)	3 (20.00%)	6 (40.00%)	1 (6.67%)
I can use it successfully every time.		1 (6.67%)		3 (20.00%)	1 (6.67%)	8 (53.33%)	2 (13.33%)
The app is tedious to work with.	3 (21.43%)	1 (7.14%)		5 (35.71%)	2 (14.29%)	3 (21.43%)	
I require written instructions to use it.		5 (35.71%)	1 (7.14%)	3 (21.43%)	1 (7.14%)	2 (14.29%)	2 (14.29%)
It is difficult to recover from mistakes.	1 (6.67%)	4 (26.67%)	1 (6.67%)	9 (60.00%)			
**Satisfaction**							
I am satisfied with it.		1 (6.67%)		1 (6.67%)	2 (13.33%)	8 (53.33%)	3 (20.00%)
It works the way I want it to work.		1 (7.14%)		1 (7.14%)	2 (14.29%)	7 (50.00%)	3 (21.43%)
The app could be better.		1 (6.67%)		1 (6.67%)	4 (26.67%)	7 (46.67%)	2 (13.33%)
The app wasn’t as satisfactory compared to other health apps	1 (6.67%)	4 (26.67%)	1 (6.67%)	8 (53.33%)	1 (6.67%)		

#### Medical Professionals

We also performed an identical usability evaluation with the 5 medical professionals. Table 2 contains the responses from the 5 medical professionals regarding trust and security, ease of use, and satisfaction.

**Table 2:** Medical professionals responses to questionnaire

Question	Strongly disagree	Disagree	Partially disagree	Neither disagree nor agree	Partially agree	Agree	Strongly agree
**Trust and security**							
When I’m connected to the Internet, I am concerned about exposing my health information to the public.					3 (60%)	2 (40%)	
I am not too concerned about what others see when I post my health-related information on the Internet.	1 (20%)	3 (60%)	1 (20%)				
This system has made me more aware of what I may be exposing to others on the network					2 (40%)	3 (60%)	
I feel safer when using the system.				1 (20%)	3 (60%)	1 (20%)	
Personal information, which I input, is managed carefully and will not be leaked to the outside.					4 (80%)	1 (20%)	
**Ease of use**							
It is easy to use.					2 (40%)	3 (60%)	
It is user-friendly.			1 (20%)		1 (20%)	3 (60%)	
It requires the fewest steps possible to accomplish what I want to do with it.						5 (100%)	
Both occasional and regular users would like it.				1 (20%)	1 (20%)	3 (60%)	
I can use it successfully every time.				2 (40%)		3 (60%)	
The app is tedious to work with.		3 (60%)		1 (20%)	1 (20%)		
I require written instructions to use it.		2 (40%)			2 (40%)	1 (20%)	
It is difficult to recover from mistakes.		2 (40%)	1 (20%)	2 (40%)			
**Satisfaction**							
I am satisfied with it.					1 (20%)	3 (60%)	1 (20%)
It works the way I want it to work.				1 (20%)		3 (60%)	1 (20%)
The app could be better.			1 (20%)	1 (20%)	2 (40%)	1 (20%)	
The app wasn’t as satisfactory compared to other health apps	1 (20%)	1 (20%)		3 (60%)			

From our trust and security results, 2 out of 5 participants (40%) were strongly concerned about exposing health information over the Internet while the rest were partially concerned. After using our app, 4 out of 5 participants (80%) felt that the personal information they entered into the app would not be leaked to the outside. Results were mainly positive about feeling safer when using the system and being more aware of what they might be exposing to others on the network. In terms of feedback, participants reported that users would not understand the key process and that it might need to be accompanied with images, for better understanding. We needed to better showcase the trivial solution of data sharing, as described in the introduction, and how our system solves the issues of the solution. Another participant reported that the 2-part encryption was ideal.

In terms of ease of use, 3 out of 5 participants (60%) agreed that the app required the fewest steps possible, in order for them to accomplish what they wanted to with the app. Results were also mostly positive, in terms of the app being user-friendly, easy to use, and the ability to use it successfully, every time. However, a few participants agreed that some form of written instructions was needed to make this app usable. Overall, medical professionals found our system satisfactory. Out of 5 participants, 4 (80%) found the app satisfactory and working in the way they wanted it to.

Similar to the potential users, 2 out of 5 medical professionals (40%) also felt that the app could have been better. For instance, most of the feedback involved improving the user interface. Doctors reported that a notification system for the app would have been very handy. The notification system could pop up or beep and alert a patient when a doctor has provided feedback. For more serious medical problems, the notification system could forward the patient’s request to an emergency unit or mental health crisis team, in the event that the doctor cannot respond out of hours. Most doctors provided positive feedback about the security of the app. One participant noted that the 2-part encryption might be frustrating for older patients, and that such a system perfectly suits teenage patients.

### Performance Tests

As a measure of performance, we tested the overhead introduced in our system, regarding the storage and retrieval of eHealth information, with simple AES encryption/decryption of similar text data. We carried out 20 test cases and measured the time taken for each test case. To carry out the tests, we used the ASUS Eee Pad Transformer Prime TF201 tablet [36] with Android OS to run our MindFeedback app. We used an HP Notebook running Windows 8 with Intel Core i5 and 4GB RAM to run the AES encryption/decryption operations and to also interface with our app, in order to retrieve performance time information from MindFeedback.

**Figure 11:**
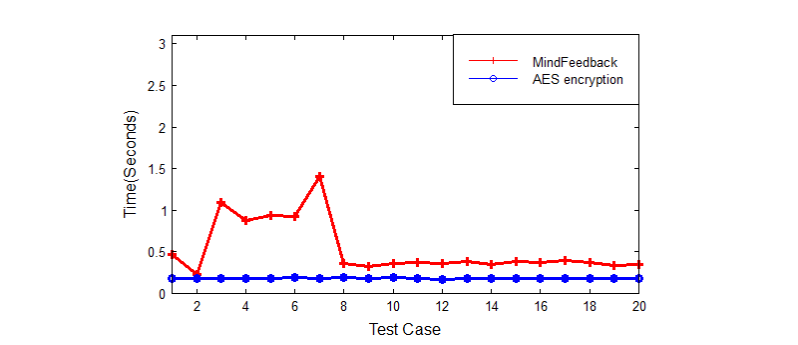
Upload Overhead.

In our performance tests, we measured the overhead introduced by our system compared with a simple AES encryption and decryption operation. We first measured the overhead introduced by uploading the patient’s health data to the cloud server. Figure 11 illustrates the results of our upload performance tests.

The diagram clearly highlights the overhead of our system compared with a simple AES encryption solution. The mean time for the simple AES symmetric encryption was 0.18 seconds, with a standard deviation of 0.006 seconds. However, the mean time for the MindFeedback tests was 0.485 seconds, with a standard deviation of 0.09 seconds. The overhead is accounted for the additional encryption of the symmetric key, followed by the partial decryption of the symmetric key through the ElGamal encryption algorithm. There was also some network latency overhead.

**Figure 12:**
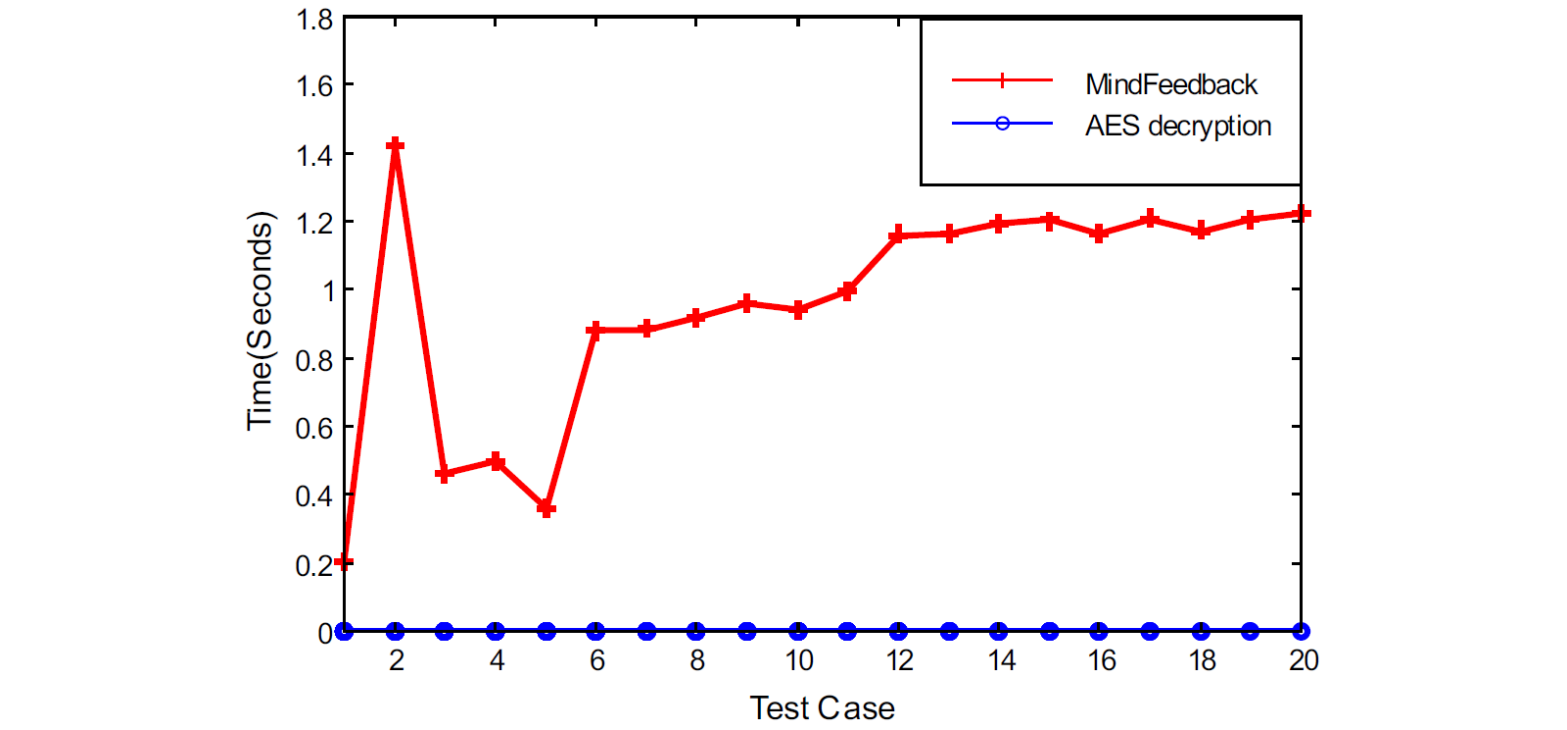
Download overhead.

We also measured the overhead introduced by our protocol for the download or retrieval of the patient’s health data. Figure 12 highlights the results of the performance tests.

As seen in the diagram, the system only had a slight overhead compared with a simple AES decryption operation. The mean time of the AES decryption tests was 0.001 seconds, with a standard deviation of 0.0003 seconds. The mean time of the MindFeedback download tests was 0.961 seconds, with a standard deviation of 0.332 seconds. Note that the patient’s encrypted key used to protect health data is first partially decrypted in the cloud server and then fully decrypted on the patient’s mobile phone. This is then followed by an AES symmetric decryption using the key on the mobile phone, thus accounting for the overhead.

### Scalability Tests

In the scalability tests, we measured the maximum load distribution that our locally deployed SOAP (Simple Object Access Protocol) Web service could handle. We used a scalability tool that made calls to the login and getData methods of our cloud service. The maximum number of threads we were able to run concurrently without the system becoming a bottleneck was 200. We carried out the tests on an HP Notebook running Windows 8 with Intel Core i5 and 4GB RAM.

**Figure 13:**
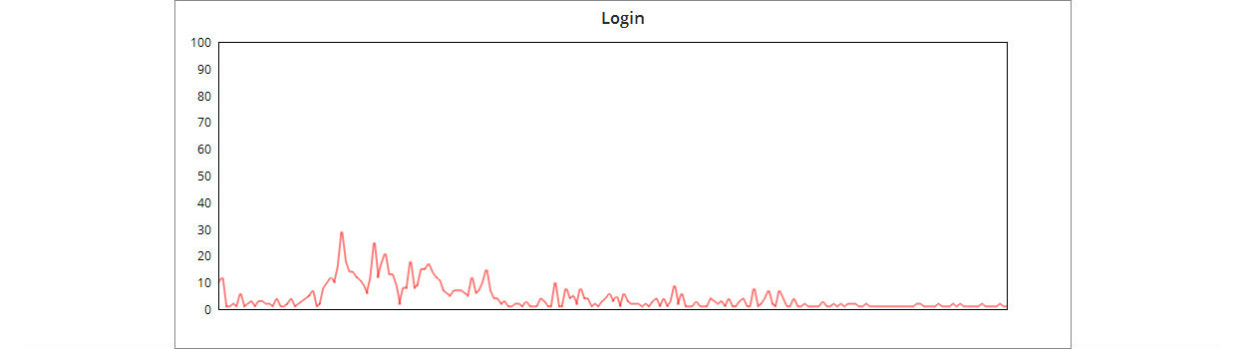
Distribution of login performance.

**Figure 14:**
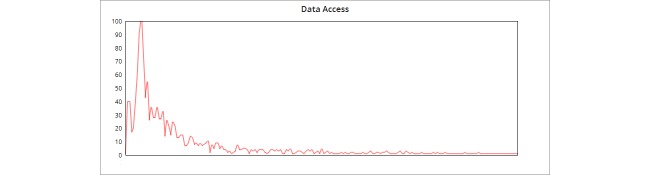
Distribution of data access from cloud performance.

See Figures 13 and 14 for our scalability distribution over the 200 threads, for both calls to log in and calls to retrieve the data from the cloud service.

The diagrams highlight the near-ideal bell curve distribution. Our system could withstand up to 200 concurrent calls to our Web service, which makes it more feasible for use in a real-world scenario.

## Discussion

eHealth applications are a fast-growing segment in the technology market; however, privacy and security issues hinder the wide-scale adoption that patients can potentially benefit from. In this paper, we presented a solution that will enable patients to share their personal health information with doctors remotely while ensuring privacy and security.

We then presented our system based on the encryption key partitioning algorithm that will enable patients and doctors to communicate with each other privately and securely. We leveraged mobile phones to provide greater convenience for patients. We carried out performance tests, usability tests, and scalability tests to show that our system is feasible to be deployed in a real-world scenario.

Our performance tests were shown to be practical to be deployed in a real-world scenario, even after it introduced a slight overhead due to our security protocol. From the usability tests, we found that many users were concerned when they shared their personal health information online and that they felt safer when using our system. A majority of participants found our app easy to use and efficient but had provided feedback that it could be better. For example, the app could have had a notification system that beeped every time a doctor sent feedback to the patient or alert the emergency unit for more serious medical problems. In terms of scalability, our system was shown to withstand up to 200 concurrent calls to our locally run Web service, thus making it feasible to be deployed in a real-world scenario.

One recommendation for further development is to remove the assumption that the doctor is trusted. That is, once the doctor is able to view the patient’s fully decrypted personal health information, she may then accidentally or inadvertently send the data to another doctor without the knowledge and/or permission of the patient. A solution could be developed to prevent unauthorized sharing of personal health information by authorized doctors. One way to do this would be to utilize an additional security token such that the health information can be viewed only if the security token is present in an authorized doctor’s device. Another way would be to perhaps encapsulate the personal health information in a secure data object and require that credentials be entered every time an authorized doctor requests access. Another recommendation for future work is to handle the scenario where a revoked doctor colludes with the social network. Currently, this will reveal the full key that will then allow the doctor to decrypt all of the personal health information stored by the patient on the social network.

## Abbreviations

ABE: attribute-based encryption
AES: Advanced Encryption Standard
ECG: electrocardiographic
OS: operating system
